# Hydrogen-Saturated Saline Protects Intensive Narrow Band Noise-Induced Hearing Loss in Guinea Pigs through an Antioxidant Effect

**DOI:** 10.1371/journal.pone.0100774

**Published:** 2014-06-19

**Authors:** Liwei Chen, Ning Yu, Yan Lu, Longjun Wu, Daishi Chen, Weiwei Guo, Lidong Zhao, Mingbo Liu, Shiming Yang, Xuejun Sun, Suoqiang Zhai

**Affiliations:** 1 Department of Otolaryngology Head and Neck Surgery, Chinese PLA General Hospital, Beijing, China; 2 Department of Otolaryngology Head and Neck Surgery, Wenzhou Medical College, Wenzhou, Zhejiang Province, China; 3 Department of Diving Medicine, Faculty of Naval Medicine, Second Military Medical University, Shanghai, China; Texas A&M University, United States of America

## Abstract

The purpose of the current study was to evaluate hydrogen-saturated saline protecting intensive narrow band noise-induced hearing loss. Guinea pigs were divided into three groups: hydrogen-saturated saline; normal saline; and control. For saline administration, the guinea pigs were given daily abdominal injections (1 ml/100 g) 3 days before and 1 h before narrow band noise exposure (2.5–3.5 kHz 130 dB SPL, 1 h). The guinea pigs in the control group received no treatment. The hearing function was assessed by the auditory brainstem response (ABR) and distortion product otoacoustic emission (DPOAE) recording. The changes of free radicals in the cochlea before noise exposure, and immediately and 7 days after noise exposure were also examined. By Scanning electron microscopy and succinate dehydrogenase staining, we found that pre-treatment with hydrogen-saturated saline significantly reduced noise-induced hair cell damage and hearing loss. We also found that the malondialdehyde, lipid peroxidation, and hydroxyl levels were significantly lower in the hydrogen-saturated saline group after noise trauma, indicating that hydrogen-saturated saline can decrease the amount of harmful free radicals caused by noise trauma. Our findings suggest that hydrogen-saturated saline is effective in preventing intensive narrow band noise-induced hearing loss through the antioxidant effect.

## Introduction

With the development of modern industry, environmental noise can cause permanent hearing loss in human. Noise-induced hearing loss (NIHL) is a sensorineural hearing deficit that is mainly caused by a strong noise stimulus. It is well known that oxidative stress within the cochlea produces large amounts of free radicals and is a major molecular mechanism underlying noise-induced deafness [1]. Moreover, the oxidative stress is also associated with age-related hearing loss and aminoglycoside-induced hearing loss [2], [3], [4].

Previous studies demonstrated that hydrogen can selectively reduce hydroxyl, peroxynitrite, and especially hydroxyl radicals, the most toxic reactive oxygen species, to inhibit oxidative stress, thus acting as an antioxidant reagent [5]. Recently, it has been found that hydrogen-saturated medium could significantly reduce antimycin A induced ROS and lipid peroxidation in hair cells, to promote hair cell survival *in vitro* [6]. Oral or intraperitoneal induction of hydrogen-rich saline can also reduce NIHL and hair cell damage *in vivo* [7], [8]. An intensive 2.5–3.5 kHz sound is one type of narrow band noise that is usually used as a warning sound and may induce a strong uncomfortable feeling which can cause severe hair cell injury and hearing loss. In this study, the effect of hydrogen-saturated saline on narrow band noise-induced hearing loss was investigated. Our results showed that hydrogen-saturated saline can act as an antioxidant reagent to protect against intensive narrow band noise-induced hearing loss.

## Materials and Methods

### 1. Animals

Healthy adult albino guinea pigs (250–300 g) of either sex were used for the experiments. Albino guinea pigs were randomly assigned to 1 of 3 experimental groups: (1) hydrogen-saturated saline (1 ml/100 g) was administered intraperitoneally 3 days (once a day) and 1 h before noise exposure (hydrogen-saturated saline group, n = 20); (2) normal saline (0.9%) was administered to the same number of guinea pigs by the same method as the control (normal saline group, n = 20); and (3) guinea pigs that did not receive any noise exposure or treatment were used as the normal control (control group, n = 15). All of the animals were bought from Vital River Laboratory Animal Technology Co. Ltd. (Beijing, China). Care and use of the animals in this study was approved by the Institutional Animal Care and Use Committee of the Chinese PLA General Hospital.

### 2. Preparation of Hydrogen-saturated Saline

Hydrogen-saturated saline was prepared as described previously [9]. Briefly, hydrogen was dissolved in normal saline for 2 h under high pressure (0.4 MPa) to supersaturation using a self-designed hydrogen-saturated water-producing apparatus. The saturated hydrogen saline was stored under atmospheric pressure at 4°C in an aluminum bag without dead volume. Hydrogen-saturated saline was freshly prepared each week to ensure a constant concentration >0.6 mM.

### 3. Noise Exposure and Procedures

The animals were exposed to a warning sound, which is a narrow band noise centered at 2.5–3.5 kHz (Sound Spotlight System 2000; China ASX Technology Co., Ltd., Beijing). The animals were exposed to the noise at a level of 130 dB SPL for 1 h. Alert, non-anesthetized animals were placed in a special restraint cage and the distance from the noise generator to both ears was set at 1 m to ensure that each ear received noise exposure of equal intensity. The noise levels were calibrated with a sound level meter (B&K type 2209) and a condenser microphone (B&K type 4136).

### 4. Auditory Brainstem Response (ABR) Measurements

Electrophysiologic measurements were made in a double-walled soundproof room. The ABR threshold value was examined in each group before noise exposure, and immediately and 1, 3, 7, and 14 days after noise exposure. Hearing was evaluated with Tucker-Davis Technologies hardware and software (TDT System III, Alachua, FL, USA). Briefly, non-anesthetized animals were placed in a special restraint cage, and ABRs were subcutaneously recorded with sterile electrode needles. The reference electrode was inserted beneath the pinna of the ear, the ground beneath the opposite ear, and the active electrode beneath the skin at the vertex. ABRs were evoked with clicks. Band-pass was filtered between 100 and 3000 Hz. Stimulus levels varied in descending steps of 5 dB. At each level, 1024 responses were averaged. The hearing threshold was confirmed until the wave III disappeared.

### 5. Measurement of the Distortion Product Otoacoustic Emission (DPOAE)

The DPOAE was examined before noise exposure, and 7 and 14 days after noise exposure. Briefly, the DPOAE was recorded using a Madsen Capella Distortion Product Otoacoustic Emission System (Madsen Capella, Taastrup, Denmark). Briefly, non-anesthetized animals were placed in a special restraint cage and the earpiece was inserted into the ear canal for DPOAE testing. Two frequency transducers were used to deliver the primary tones, f_l_ and f_2_, to the ear canal through flexible tubes connected to the earpiece. The f_2_/f_1_ ratio was set at 1.2. The DPOAE input/output functions were measured at f_2_ frequencies of 500, 750, 1000, 1500, 2000, 3000, 4000, 6000, and 8000 Hz with the same intensity of L2/L1 = 55/65 dB.

### 6. Scanning Electron Microscopy (SEM)

On the 14th day after noise trauma, the animals were sacrificed by decapitation and the cochleae were removed. Round and oval windows of the cochleae were perforated with a sharp pick. The peri-lymphatic space was perfused with 2.5% glutaraldehyde in 0.1 M PBS, and each specimen was placed in glutaraldehyde solution overnight. The cochleae were washed three times with PBS. The specimens were fixed with 1% OsO_4_ for 15 min. The samples were then washed in PBS three times. The tissues were serially dehydrated in 50%, 70%, 90%, and 100% ethanol. After critical point drying with CO_2_ and sputter-coating with gold, the tissues were examined using a Hitachi S-4800 scanning electron microscope.

### 7. Histologic Analysis of the Cochlea

On the 7th day after noise trauma, the animals were sacrificed and the cochleae were removed. The round window membrane was opened, and succinate dehydrogenase (SDH) staining solution (1∶1∶2 0.2 M sodium succinate, 0.2 M PBS [pH 7.6], and 0.1% tetranitro-blue tetrazolium) was gently perfused through the round and oval windows. Subsequently, the cochleae were immersed in SDH solution for 60 min at 37°C. The specimens were later fixed with 4% formalin for 24 h. The sensory epithelium was removed from the cochlea in 0.01 M PBS and mounted in glycerin on glass slides as surface preparations. The sensory epithelium was viewed with a light microscope (Olympus BX51, Tokyo, Japan) and photographed with a highly sensitive camera (Olympus DP-72) at 400× magnification using DP2-BSW software.

### 8. Detection of Lipid Peroxidation (LPO), Malondialdehyde (MDA), and Hydroxyl (·OH) Levels in the Cochlea

The frozen cochlear tissues were harvested and homogenized immediately 5 times at 5000 rotations per min for 30 sec by a high-speed homogenizer in an ice bath in 10 volumes of physiologic saline. The homogenates were centrifuged at 6000 G for 10 min at 4°C. The levels of LPO, MDA, and ·OH in the supernatant were detected using the corresponding assay kits according to the manufacturer’s recommendations. Each sample consisted of 10 cochleae from 5 guinea pigs.

### 9. Statistical Analysis

All data are expressed as the mean ± standard deviation (SD). Data were analyzed using an unpaired t-test (Student’s t-test) for two groups and a one-way ANOVA with Bonferroni *post hoc* tests for multiple comparisons, as noted in the figure legends (SPSS 13.0 software). P<0.05 were deemed statistically significant.

## Results

### 1. ABR

**Figure 1:**
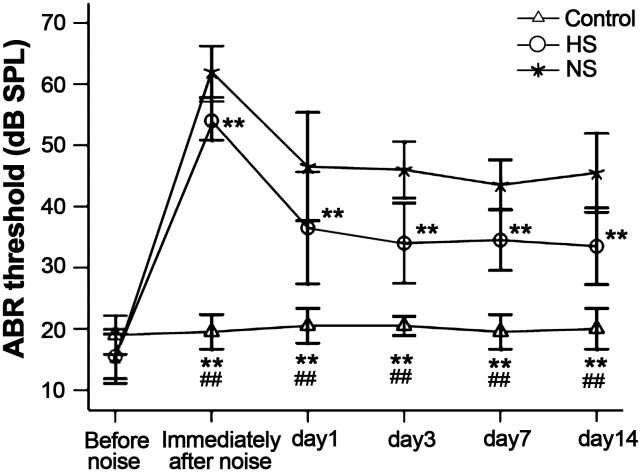
Noise-induced ABR threshold shifts. Immediately after noise exposure (130 dB SPL for 1 h), the ABR threshold of the hydrogen-saturated saline and normal saline groups was 54 dB and 62 dB, respectively. The ABR was subsequently tested 14 days after noise exposure. The hydrogen-saturated saline group had less of an ABR threshold shift compared to the normal saline group, 3, 7, and 14 days after noise exposure. HS: hydrogen-saturated saline group; NS: normal saline group. **P<0.01: vs. NS; ##P<0.01: control vs. HS.

Before noise exposure, ABR tests confirmed that guinea pigs had normal hearing function. Immediately after intensive narrow band noise exposure (130 dB SPL for 1 h), the guinea pigs in the hydrogen-saturated saline group had an average ABR threshold shift of 54 dB SPL. The guinea pigs in the normal saline group had an average ABR threshold shift of 62 dB SPL. The ABR was subsequently tested 1, 3, 7, and 14 days after noise exposure. The results showed that guinea pigs in the hydrogen-saturated saline group had less of an ABR threshold shift compared to the guinea pigs in the normal saline group (Fig. 1).

### 2. DPOAE

**Figure 2:**
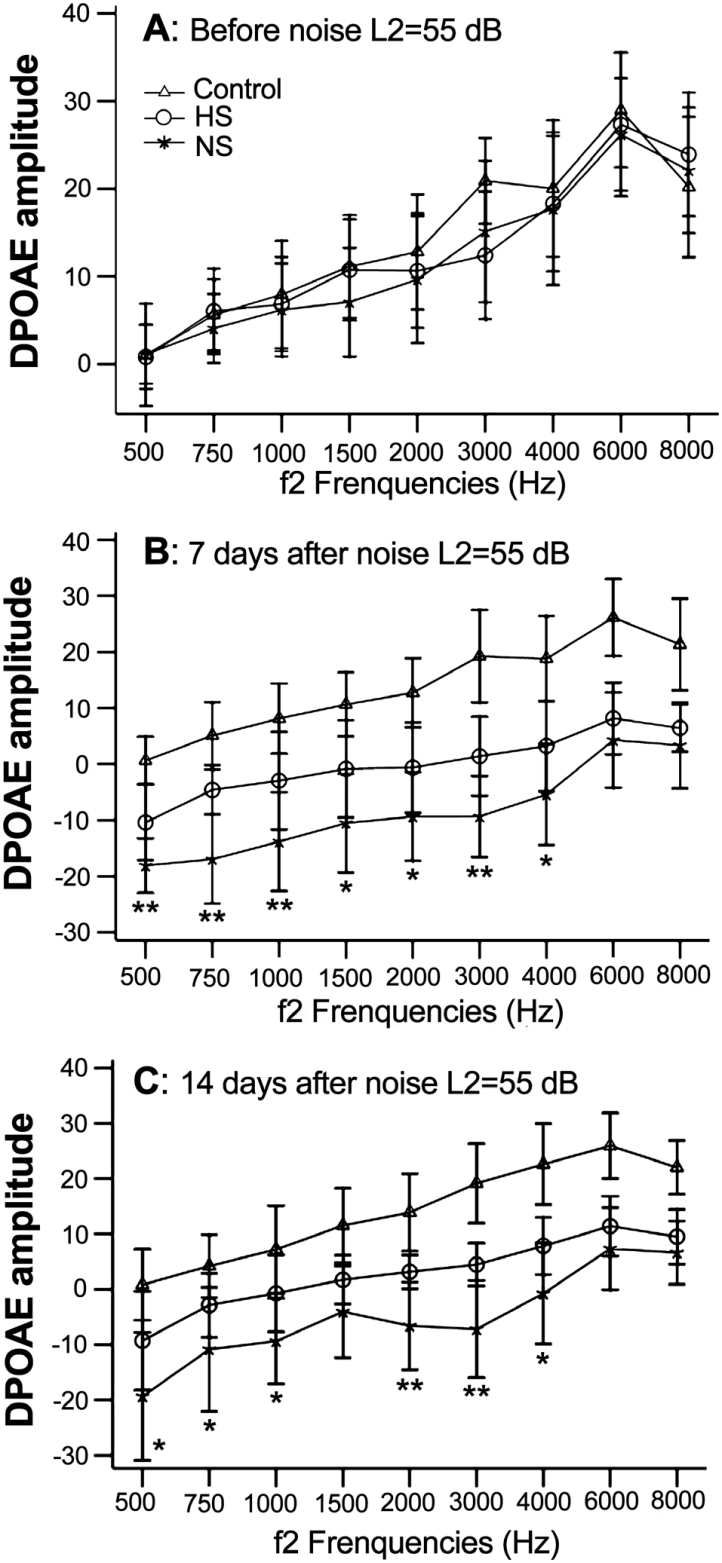
DPOAE amplitudes were detected before and after noise exposure. (A) The average DPOAE amplitudes were normal in the three groups before noise exposure. (B) The average DPOAE amplitudes were markedly decreased 7 days after noise exposure. There were significant differences in the mean DPOAE amplitudes between the hydrogen-saturated and normal saline groups at frequencies of 0.5–4 kHz. (C) 14 days after noise exposure, the DPOAE amplitudes of the hydrogen-saturated saline group were significantly better than the normal saline group, especially at frequencies of 0.5, 0.75, 1, 2, 3, and 4 kHz. HS: hydrogen-saturated saline group; NS: normal saline group; *P<0.05, **P<0.01: hydrogen-saturated saline group vs. normal saline group.

The DPOAE amplitudes were tested to assess the noise damage to the outer hair cells (Fig. 2). After noise exposure, the amplitude of DPOAE at 1–8 kHz significantly decreased in the groups. Seven days after noise damage, the guinea pigs in the hydrogen saline group had less of a decrease in DPOAE amplitudes at frequencies of 0.5–4 kHz compared to the guinea pigs in the control group. Fourteen days after noise exposure, the DPOAE amplitude at frequencies of 0.5, 0.75, 1, 2, 3, and 4 kHz for the guinea pigs in the hydrogen-saturated saline group were significantly higher than the normal saline group.

### 3. Scanning Electron Microscopy

**Figure 3:**
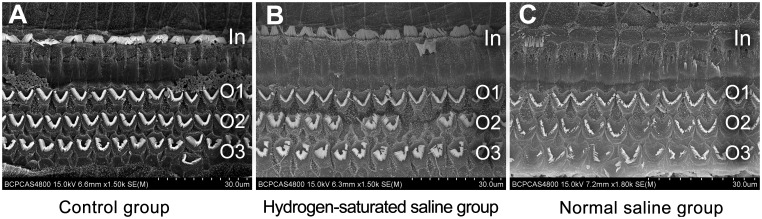
SEM findings after noise exposure. Fourteen days after noise trauma, scanning electron microscopy was performed. After pre-treatment with hydrogen-saturated saline, damage to the stereocilia of hair cells decreased significantly compared with the normal saline group. (A) Control group. (B) Hydrogen-saturated saline group. (C) Normal saline group. In: inner hair cell layer. O1–3: outer hair cell layers.

Scanning electron microscopy showed that the morphology of hair cells was minimally damaged in the guinea pigs treated with hydrogen-saturated saline. In contrast, the inner hair cell stereocilia were missing in some regions in the normal saline group, and the outer hair cell stereocilia were severely damaged by noise trauma with a large number prostrate or missing. The damage was more evident in the 3rd row of outer hair cells in the basal turns of the cochlea (Fig. 3). Thus, the scanning electron microscopy results are also consistent with the ABR and DPOAE results.

### 4. Succinate Dehydrogenase (SDH) Staining

**Figure 4:**
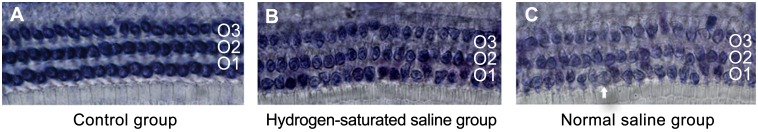
Surface preparation of the organ of Corti in the basal turn stained with SDH. Seven days after noise exposure, the cochleae were stained with SDH. The staining and the morphology of the hair cells in the hydrogen-saturated saline group were superior to the normal saline group. (A) Control group. (B) Hydrogen-saturated saline group. (C) Normal saline group. O1–3: outer hair cell layers. “↑”: dropsical hair cells.

Seven days after noise exposure, guinea pigs were sacrificed for immunohistochemical examination. The cochleae were stained with SDH. The hair cells of the hydrogen-saturated saline group were minimally damaged, and the staining of the SDH was also stronger than the normal saline group. Instead, the hair cells of the normal saline group had a large percentage of damage. Interestingly, the damaged hair cells of the normal saline group were mostly dropsical, with a minimal percentage of missing cells (Fig. 4).

### 5. Free Radical Detection

**Figure 5:**
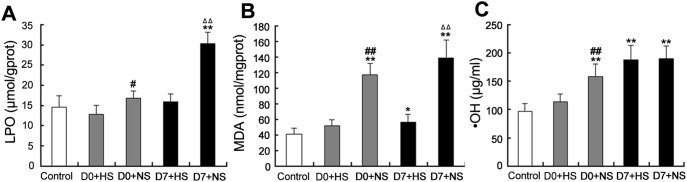
Free radicals in the cochleae were detected after noise exposure. (A, B) After noise exposure, the LPO and MDA levels of the normal saline group were significantly higher than the hydrogen-saturated saline group. (C) Immediately after noise exposure, the content of ·OH in the cochleae of the normal saline group was significantly higher than the hydrogen-saturated saline group. The difference was abolished by the 7th day after noise exposure. D0/D7+HS: immediately/7days after noise exposure of hydrogen-saturated saline group. D0/D7+NS: immediately/7days after noise exposure of normal saline group. *P<0.05, **P<0.01: vs. control. #P<0.05, ##P<0.01: D0+NS vs. D0+HS. ΔP<0.05, ΔΔP<0.01: D7+NS vs. D7+HS.

The free radical levels were measured in the cochleae before noise exposure, and immediately and 7 days after noise exposure (Fig. 5). Immediately and 7 days after noise exposure, the levels of expression of LPO and MDA were significantly lower in the cochleae of the hydrogen-saturated saline group as compared to the normal saline group. Immediately after noise exposure, the levels of OH in the guinea pigs pre-treated with hydrogen-saturated saline were significantly lower than the guinea pigs pre-treated with normal saline. Interestingly, the difference was abolished 7 days after noise exposure.

## Discussion

Direct mechanical damage and indirect metabolic damage are recognized as major causes of noise-induced deafness. The excessive expression of ROS after noise trauma in the cochlea is recognized as the most important factor causing cochlear damage [10]. Lim and Melnick [11] first proposed that noise causes metabolic changes in the cochlea, damages the inner ear structure, and results in deafness. Yamane et al. [12] first discovered that free radicals are generated in inner ear tissues after noise exposure. Subsequent studies have confirmed that excessive production of free radicals is a major cause of cochlear hearing loss [13], [14], [15]. It has also been reported that free radicals are widely generated in various cochlear tissues, including vascular vessels, outer hair cells, spiral ganglion cells, and supporting tissues, and the expression of free radicals after noise exposure can persist for 2 weeks [16], [17], [18]. Thus, preventing the generation of free radicals and the application of antioxidant drugs are critical in reducing NIHL. A series of studies have shown that antioxidants, such as R-phenylisopropyladenosine (R-PIA), glutathione monoethylester, salicylate, acetyl-L-carnitine (ALCAR), and N-L-acetylcysteine (NAC), as well as free radical scavengers (vitamins A, C and E), can prevent hair cell damage and hearing loss induced by noise trauma [19], [20], [21], [22], [23]; however, these drugs have limited protective effects as they do not cross the lipid membrane barriers, thus inefficiently scavenging free radicals [1].

In recent years hydrogen has been used as a new reagent of antioxidants to protect tissues in various organs [24]. Studies have shown that there are several benefits to using hydrogen as a potential antioxidant; specifically, hydrogen can effectively neutralize ·OH in living cells, easily diffuse into the cytosol, mitochondria, or nucleus through biomembranes, and quickly spread to the target area to achieve the goal of highly efficient anti-free radical effects [5]. These characteristics of hydrogen suggest that hydrogen may cross the blood-labyrinth barrier to reach the cochlear hair cells, thus making hydrogen an ideal cochlear antioxidant.

It has been shown that hydrogen-saturated medium can significantly reduce antimycin A-induced ROS, and subsequent LPO in hair cells *in vitro* and increased hair cell survival [6], providing a theoretic basis for research on the prevention and treatment of NIHL with hydrogen. In the present study, we found that pre-treatment with hydrogen-saturated saline not only reduced temporary hearing threshold shifts, but also prevented permanent hearing threshold shifts. Fourteen days after noise exposure, the hydrogen-saturated saline group showed better ABR and DPOAE results. After hearing testing, electron microscopy showed that the stereocilia of hair cell were scattered, lying prostrate, and even missing in the normal saline group, whereas the stereocilia were minimally damaged in the hydrogen-saturated saline group. The results of SDH staining were also superior in the hydrogen-saturated saline group to the normal saline group, suggesting that hydrogen protected the function of mitochondria and maintained a normal tricarboxylic acid cycle, thus protecting noised-induced hair cell damage in the cochlea. These morphologic results are consistent with the results of audiologic testing.

It has been reported that a nearly 4-fold increase in ·OH radicals in outer hair cells within 1–2 h of noise exposure [14]. MDA and LPO levels increase after noise trauma is detected [25], [26]. To verify if the protective effect of hydrogen-saturated saline against noise-induced hearing loss is due to its antioxidant effects, we examined the free radicals before noise exposure, and immediately and 7 days after noise exposure. We found that immediately and 7 days after noise exposure, cochlear LPO and MDA content in the normal saline group were higher than the hydrogen-saturated saline group. Immediately after noise exposure, the cochlear ·OH content in the normal saline group was also significantly higher than the hydrogen-saturated saline group. Seven days after noise exposure, however, the difference was not statistically significant. We speculate that hydrogen can decrease the amount of harmful free radicals caused by noise trauma. We cannot provide direct experimental evidence demonstrating how hydrogen removes these harmful free radicals; however, it is clear that hydrogen is an antioxidant. Our experiments show that hydrogen has a significant protective effect against intensive narrow band noise-induced hair cell damage, and the protective effect of hydrogen is via an antioxidant mechanism.

Currently, there are only two studies involving hydrogen protection on NIHL [7], [8]. In those studies, the intensity of noise used was 115 dB SPL, which was markedly less than the 130 dB SPL used in present study. The administration time of hydrogen was significantly longer (6 and 14 days), as compared to 3 days and 1 h before noise exposure in the current study. Our experiments showed that a small amount of hydrogen and a short period of administration is sufficient to protect intensive narrow band noise-induced hearing loss in guinea pigs. Future studies are needed to optimize the time points and the amount of administration, even the signal pathway to deepen our understandings of the antioxidant effect of hydrogen in the cochlea.
